# Co‐Atomic Interface Minimizing Charge Transfer Barrier in Polytypic Perovskites for CO_2_ Photoreduction

**DOI:** 10.1002/advs.202410437

**Published:** 2025-01-10

**Authors:** Fengyi Zhong, Jianping Sheng, Chenyu Du, Ye He, Fengying Zhang, Yanjuan Sun, Ying Zhou, Fan Dong

**Affiliations:** ^1^ School of Resources and Environment University of Electronic Science and Technology of China Chengdu 611731 China; ^2^ Institute of Fundamental and Frontier Sciences University of Electronic Science and Technology of China Chengdu 611731 China; ^3^ School of New Energy and Materials Southwest Petroleum University Chengdu 610500 China

**Keywords:** charge transfer kinetics, CO_2_ photoreduction, co‐interface, photocatalysis, polytypic nanocrystals

## Abstract

Heterojunctions, known for their decent separation of photo‐generated electrons and holes, are promising for photocatalytic CO_2_ reduction. However, a significant obstacle in traditional post‐assembled heterojunctions is the high interfacial barrier for charge transfer caused by atomic lattice mismatch at multiphase interfaces. Here, as research prototypes, the study creates a lattice‐matched co‐atomic interface within CsPbBr_3_‐CsPb_2_Br_5_ polytypic nanocrystals (113‐125 PNs) through the proposed in situ hybrid strategy to elucidate the underlying charge transfer mechanism within this unique interface. Compared to CsPbBr_3_ nanocrystals, the 113–125 PNs exhibit a remarkable 3.6‐fold increase in photocatalytic CO_2_ reduction activity (173.3 µmol^−1^ g^−1^ within 5 h). Furthermore, Kelvin probe force microscopy results reveal an increase in the built‐in electric field within this lattice‐matched co‐atomic interface from 43.5 to 68.7 mV, providing a stronger driving force for charge separation and directional migration. Additionally, ultrafast transient absorption spectroscopy uncovers the additional charge carrier transfer pathways across this lattice‐matched co‐atomic interface. Thus, this unique co‐atomic interface significantly promotes the interfacial electronic coupling and mitigates the charge transfer barrier, thus facilitating efficient charge separation and transfer. These insights underscore the importance of interfacial structure in heterojunction design and comprehending the intricate interplay between interface and carrier dynamics.

## Graphical abstract

The study develops CsPbBr_3_‐CsPb_2_Br_5_ polytypic nanocrystals with a lattice matched co‐atomic interface, achieving a 3.6‐fold increase in photocatalytic CO_2_ reduction compared to CsPbBr_3_ nanocrystals. The lattice‐matched interface reduces charge transfer barriers and enhances efficiency, highlighting the importance of precise interfacial design in optimizing heterojunctions for photocatalytic applications.

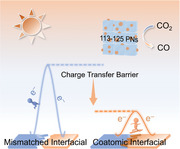

## 1 Introduction

The increasing levels of atmospheric CO_2_ and their detrimental impact on the global climate necessitate the development of sustainable technologies to mitigate CO_2_ emissions. Photocatalytic CO_2_ reduction, using solar energy to drive the conversion of CO_2_ into valuable chemicals or clean fuels, offers an attractive solution.^[^ ^1^ ^]^ Among various semiconductor materials, the CsPbBr_3_ perovskite nanocrystals have gained considerable attention for their excellent optoelectronic properties, including their tunable bandgap and high electron‐hole mobility. However, the rapid recombination of photoinduced charges restricts its overall photocatalytic efficiency. To further enhance CsPbBr_3_ perovskite photocatalytic performance, increasing the efficiency of charge separation is an urgent issue.^[^ ^2^ ^]^

In the pursuit of significantly amplifying the efficiency of charge separation in CsPbBr_3_ perovskite nanocrystals, the implementation of heterojunction hybridization emerges as a potent strategy.^[^ ^3^ ^]^ The heterojunction construction induces alterations in the electronic structure and band alignment of the different materials.^[^ ^4^ ^]^ In the heterojunction, the mechanism of action involves the creation of a potential gradient that facilitates the directional movement of charge carriers and their spatial separation. The introduction of heterointerfaces fosters the synergistic interactions between different components, promoting charge transfer kinetics and reducing charge recombination losses.^[^ ^5^ ^]^ This chemical modulation at the heterojunction interface further amplifies the overall efficiency of photocatalytic reactions, making the heterojunction a compelling strategy for tailoring the chemical behavior of CsPbBr_3_ perovskite nanocrystals. However, it should be acknowledged that there are significant limitations associated with heterojunction design, mainly arising from lattice atom mismatch in conventional heterointerfaces. The mismatch introduces high charge transfer barriers, imposing constraints on the effective separation of electrons and holes. Therefore, this limitation must be overcome to unlock the full potential of charge separation in CsPbBr_3_ perovskite nanocrystals.

**Figure 1:**
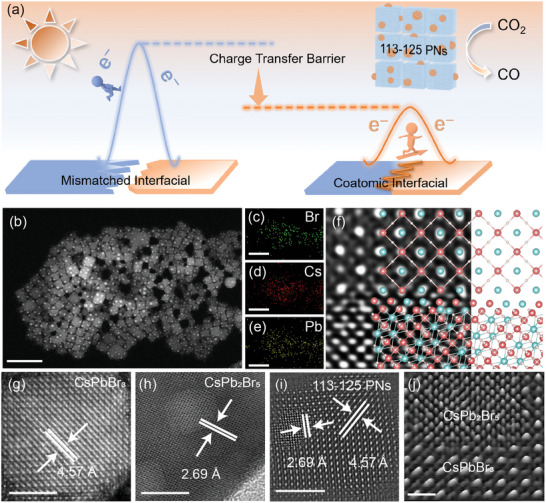
Microscopy characterizations during in situ 113–125 PNs formation. a) Schematic diagram of 113–125 PNs co‐atomic interface reducing charge transfer barrier and improving photocatalytic CO_2_ reduction activity. b) Representative HAADF‐STEM image. c–e) Element mapping, and f) enlarged view of the interface and atomic simulation diagram (Cs = blue, Pb = red, Br = white) in 113–125 PNs. g) HAADF‐STEM image of CsPbBr_3_. h) High‐resolution TEM images of CsPb_2_Br_5_. i) Lattice‐labeled HAADF‐STEM images and j) partially enlarged 3D model diagram of 113–125 PNs. The scale bars in (b–e) are 50 nm, (g–i) are 5 nm, and (j) is 1 nm.

The concept of polytypic nanocrystals is poised to become a promising strategy for overcoming the significant charge transfer barriers arising from lattice atom mismatch in traditional heterojunction interfaces.^[^ ^6^ ^]^ Polytypic nanocrystals consist of two or more phases that share the same crystal structure but possess different lattice constants and stacking sequences. Various crystal lattice structures exist within the crystals, which were dynamically formed during the crystal growth process.^[^ ^7^ ^]^ Such diverse lattice structures can significantly alleviate the charge transfer barriers at heterojunction interfaces, thereby enhancing the separation efficiency of photo‐generated electrons and holes. One intriguing polytypic nanocrystal system is the CsPbBr_3_‐CsPb_2_Br_5_ polytypic nanocrystals (113–125 PNs), which exhibit unique electronic properties and can facilitate efficient charge separation and transfer. Electrons and holes are generated in CsPbBr_3_ upon photoexcitation due to its favorable band structure. The built‐in electric field at the interface between CsPbBr_3_ and CsPb_2_Br_5_ assists in the directional transport of charges, preventing their recombination and improving charge separation efficiency. Additionally, the heterojunction structure directs the photo‐generated charge carriers toward the interfacial region, where the CO_2_ reduction occurs (as shown in the schematic diagram in **Figure** **1**a).^[^ ^8^ ^]^ However, how these polytypic nanocrystals contribute to alleviating the charge transfer barriers at heterojunction interfaces and promoting charge separation efficiency remains unknown, which is a universal challenge.

In this study, we synthesized 113–125 PNs through an in situ hot‐injection method. It has been demonstrated that polytypic nanocrystals can break the limitations in charge separation efficiency caused by lattice atom mismatch at conventional heterojunction interfaces. High‐Angle Annular Dark‐Field Scanning Transmission Electron Microscopy (HAADF‐STEM) reveals the coexistence of atomic structures at the co‐atomic interface of 113–125 PNs. Kelvin probe force microscopy (KPFM) experiments further confirm that the charges can efficiently transfer through the built‐in electric field at the co‐atomic interface between the CsPbBr_3_ and CsPb_2_Br_5_ phases. Ultrafast femtosecond transient absorption spectroscopy (TAS) provides an in‐depth analysis of the carrier separation process within this co‐atomic interface from the perspective of electron dynamics. The polytypic nanocrystal co‐atomic interfaces provide additional sites for carrier generation, separation, and transfer. As a result, the 113–125 PNs achieved an excellent photocatalytic CO_2_‐to‐CO yield of 173.3 µmol^−1^ g^−1^ within 5 h simulated solar irradiation in a gas‐solid photocatalytic system, which was 3.63 times higher than pure CsPbBr_3_ perovskite nanocrystals. All experimental outcomes indicate that the unique structure of the co‐atomic interface within 113–125 PNs contributes to efficient charge separation and suppression of charge recombination, thereby enhancing the photocatalytic CO_2_ reduction performance. This work developed a heterojunction with the co‐atomic interface from a different pattern and emphasizes the importance of understanding the complex interactions between carrier dynamics and interface lattice structure in photocatalytic reactions.

## 2 Results and Discussion

### 2.1 Construction and Characterization of Co‐Atomic Interface

**Figure 2:**
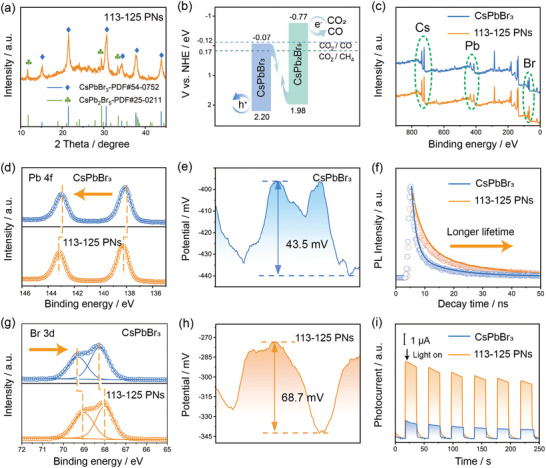
Electron transfer characteristic of polytypic nanocrystals. a) XRD patterns of 113–125 PNs; b) Diagrams of the relative band‐edge positions of CsPbBr_3_ and CsPb_2_Br_5_. The band structure and XPS spectra of c) total survey, d) Pb 4f, g) Br 3d of CsPbBr_3_, and 113–125 PNs; the line scanning surface potential profile of e) CsPbBr_3_ and h) 113–125 PNs. f) Time‐resolved fluorescence spectra. i) Photocurrent density of CsPbBr_3_ and 113–125 PNs.

The co‐atomic interface within 113–125 PNs is synthesized in situ through a controlled two‐step reaction. Initially, CsPbBr_3_ nanocrystals are synthesized using a typical hot‐injection method,^[^ ^9^ ^]^ followed by the introduction of a PbBr_2_ precursor, which leads to the nucleation and growth of CsPb_2_Br_5_ domains on the surface of CsPbBr_3_. Figure 1g shows the morphology of the synthesized CsPbBr_3_ nanocrystals characterized by aberration‐corrected high‐resolution HAADF‐STEM, demonstrating that the CsPbBr_3_ nanocrystals display a typical perovskite cube shape. The polytypic structures for 113–125 PNs are constructed by the partial phase transformation of the CsPbBr_3_ crystal phase after cooling and repeat heating to 210 °C. And 113–125 PNs derived from CsPbBr_3_ nanocrystals, which inherit the main atomic arrangement and basic cubic morphology of CsPbBr_3_ phases (Figure 1b). Concurrently, adjusting the recrystallization reaction time and temperature can control the degree of in situ growth of CsPb_2_Br_5_ particles on CsPbBr_3_ cubes.^[^ ^10^ ^]^ Transmission Electron Microscope (TEM) images indicate a progressive growth of the CsPb_2_Br_5_ phase over time (Figure S1, Supporting Information). As the reaction time extends, the initial nanocrystal structure of CsPbBr_3_ undergoes a gradual collapse. Ultimately, the CsPb_2_Br_5_ lamellar structure is fully established, resulting in the loss of the advantageous polytypic nanocrystal configuration, as depicted in Figure 1h. Furthermore, we conducted crystal spacing analysis on CsPbBr_3_, CsPb_2_Br_5_, and 113–125 PNs, respectively, demonstrating that 113–125 PNs is composed of phases CsPbBr_3_ and CsPb_2_Br_5_, with lattice spacings of 4.57 and 2.69 Å respectively (Figure 1i), the 4.57 Å corresponds to the (1 1 0) crystal plane, and the 2.69 Å corresponds to the (3 1 0) crystal plane. To confirm the common crystal plane of 113–125 PNs, we identified the corresponding perpendicular crystal planes, and ultimately confirmed that the common plane of 113–125 PNs is the (−1 3 0) plane of CsPb_2_Br_5_ and the (1 ‐1 0) plane of CsPbBr_3_. And energy dispersive spectrometer element mapping (Figure 1c–e) was utilized to characterize the element distribution in one randomly selected 113–125 PNs. The general plane spectrogram only detects uniformly distributed Cs, Pb, and Br elements in the samples. The matching of lattice stripes and mapping proves that 113–125 PNs are completely composed of the two phases CsPbBr_3_ and CsPb_2_Br_5_. To gain a deeper understanding of the interfaces in 113–125 PNs, we utilize the representative partially enlarged 3D model diagram to analyze the heterointerfaces within 113–125 PNs (Figure 1j). CsPbBr_3_ and CsPb_2_Br_5_ are arranged in an interleaved manner at the interface. We further established theoretical models for the CsPb_2_Br_5_ and CsPbBr_3_ phases within 113–125 PNs heterointerfaces (Figure 1f), and the corresponding results revealed that identical atomic arrangements exist at the interface layer. The growth of CsPb_2_Br_5_ on the CsPbBr_3_ structural domain shares a common boundary cationic structure with Cs^+^ or Pb^2+^ ions to ensure lattice compatibility. This unique boundary is further evidenced by the presence of identical atoms at the interface of the two phases, collectively contributing to the lattice‐matched co‐atomic crystal structures of CsPb_2_Br_5_ and CsPbBr_3_ within 113–125 PNs heterointerfaces.^[^ ^11^ ^]^ These shared atomic configurations between the two coexisting phases will construct a co‐atomic interface pathway within the 113–125 PNs, facilitating a tighter integration of the polytypic phases and establishing a more efficient conduit for charge transfer. The presence of identical atoms is a crucial component of polytypic nanocrystals, effectively alleviating the charge transfer barriers at heterojunction interfaces and thereby enhancing the efficiency of charge separation between photo‐generated electrons and holes.^[^ ^12^ ^]^ In addition to morphological features, we also confirmed the compositional structure of 113–125 PNs through X‐ray diffraction (XRD) and X‐ray photoelectron spectroscopy (XPS). The XRD pattern (**Figure** **2**a) of the 113–125 PNs exhibits a typical CsPb_2_Br_5_‐like diffraction pattern with enhanced peaks and retains the characteristic peak of CsPbBr_3_, demonstrating the existence of CsPbBr_3_ and CsPb_2_Br_5_ structures and proving the polytypic structure of the obtained 113–125 PNs.^[^ ^13^ ^]^ In addition, no other elements are detected in the XPS total survey (Figure 2c), which indirectly indicates that 113–125 PNs are homogeneous composed of CsPbBr_3_ and CsPb_2_Br_5_. All the results demonstrate that the lattice‐matched co‐atomic interface has been successfully in situ created within 113–125 PNs.

### 2.2 Charge Transfer Mechanism Across Co‐Atomic Interface

To investigate the charge transfer relationship between the phases of 113–125 PNs and demonstrate the prominent contribution of polytypic in photocatalysis, we conducted a comprehensive exploration of the interfacial electronic interaction between the CsPbBr_3_ and CsPb_2_Br_5_ phase within the 113–125 PNs at the atomic level. High‐resolution XPS was employed initially to compare the respective chemical states of CsPbBr_3_ nanocrystals and 113–125 PNs to investigate the chemical interaction and electron transfer between the two phases. The binding energies of Pb 4f_5/2_ and Pb 4f_7/2_ of CsPbBr_3_ nanocrystals are located at 143 and 138.1 eV, respectively (Figure 2d). In comparison, the binding energy peaks of 113–125 PNs shift positively to 143.1 and 138.3 eV. The Br 3d_1/2_ and Br 3d_3/2_ signals of 113–125 PNs shift negatively from 68.3 and 69.3 eV of CsPbBr_3_ nanocrystals to 67.9 and 69.0 eV, respectively (Figure 2g). In 113–125 PNs, the increase in Pb binding energy indicates a decrease in the electron density around Pb, with these electrons transferring to Br. Due to Br higher electronegativity, Br is more inclined to accept these electrons. The change in electron binding energy is attributed to the lattice matching between CsPbBr_3_ and CsPb_2_Br_5_, which causes lattice distortion at the interface and leads to changes in the electronic environment around Pb and Br. The electronic localization function (ELF) also shows that electrons accumulate at the Br atom at the 113–125 PNs interface (Figure S2, Supporting Information), which is consistent with the results of XPS. Besides, an intrinsic electric field is formed at the co‐atomic interface between the CsPbBr_3_ and CsPb_2_Br_5_ phase, resulting in energy level modulation at the interface, further promoting spatial charge separation. The band positions of CsPbBr_3_ and CsPb_2_Br_5_ can then be outlined in Figure 2b using the calculated bandgap values (Figure S3, Supporting Information) and the measured valence band energy (Figure S4, Supporting Information). Through Density Functional Theory (DFT) calculations, we obtained the work functions of CsPbBr_3_ and CsPb_2_Br_5_ as 5.517 and 4.367 eV, respectively. The larger work function of CsPbBr_3_ means that electrons in 113–125 PNs are less likely to escape from CsPbBr_3_, altering the conventional charge transfer mechanism and resulting in the formation of a typical S‐scheme heterojunction rather than a traditional type‐I heterojunction. The S‐scheme heterojunction indicates that the formation of CsPb_2_Br_5_ on the CsPbBr_3_ substrate constructs a lattice‐matched co‐atomic interface, which can lower the charge transfer barrier and facilitate the transfer of electrons within CsPbBr_3_. Theoretically, this makes 113–125 PNs more suitable for photocatalytic CO_2_ reduction.

The KPFM technique enables the direct visualization and quantitative analysis of the local surface potential of nanomaterials, providing valuable insights into their charge carrier behavior and charge transfer mechanisms. We employed KPFM testing to systematically study the transport behavior of photo‐generated charges in 113–125 PNs. Surface potential distribution analysis using KPFM can demonstrate the strength of the charge transfer driving force.^[^ ^14^ ^]^ Figures S5 and S6 (Supporting Information) display the 3D surface potential distribution maps of CsPbBr_3_ and 113–125 PNs. The color scale represents the surface potential values, where purple colors indicate high potential values and red colors represent low potentials. The analysis of the 3D surface potential distribution indicates a relatively uniform potential distribution across the surfaces of both CsPbBr_3_ and 113–125 PNs. Besides, corresponding line scanning surface potential profiles reveal that the surface potential of CsPbBr_3_ and 113–125 PNs was measured to be 43.5 and 68.7 mV, respectively (Figure 2e,h). This different surface potential demonstrates that 113–125 PNs possess a stronger interfacial electric field, which can act as a powerful driving force to promote charge separation and directed migration across the highly efficient co‐atomic interface.^[^ ^15^ ^]^ The relatively uniform surface potential distribution of CsPbBr_3_ suggests minimal charge accumulation and charge transfer within the nanocrystals. This behavior is attributed to the absence of an internal interfacial electric field, thereby resulting in ineffective charge carrier separation at the CsPbBr_3_ surface. In contrast, 113–125 PNs with a lattice‐matched co‐atomic interface not only serve as skeletal support to maintain interface stability and reduce charge transfer barriers between the two phases, but also, more importantly, create an internal electric field at the interface enhances charge separation and promotes interfacial charge transfer.^[^ ^16^ ^]^ In summary, the stronger internal electric field at the co‐atomic heterojunction interface of 113–125 PNs results in a more significant charge accumulation region, facilitating more effective charge separation and transfer processes, which is crucial for enhancing CO_2_ photocatalytic reduction.

### 2.3 Dynamic Analysis of Photoelectrochemical Properties

We further characterized the photoelectric properties to gain deeper insight into the structure‐activity relationship between 113–125 PNs photoelectrical properties and photocatalytic activity. First, light‐harvesting ability is necessary for photocatalytic performance. The absorption spectra of the synthesized CsPbBr_3_ nanocrystals and 113–125 PNs were characterized to assess the light‐harvesting capability by UV–vis diffuse reflectance spectra (UV–vis DRS) absorption spectroscopy in Figure S7 (Supporting Information).^[^ ^17^ ^]^ Fortunately, the 113–125 PNs maintain a similar infrastructure to the CsPbBr_3_ nanocrystals. Both the CsPbBr_3_ and 113–125 PNs spectra exhibit similar absorption in the visible region, qualifying polytypic 113–125 PNs as promising photocatalytic materials. In addition, nanosecond‐level time‐resolved fluorescence decay spectroscopy was employed to investigate the electron transfer dynamics in CsPbBr_3_ nanocrystals and 113–125 PNs (Figure 2f). The results indicate that the carrier lifetime of 113–125 PNs is significantly longer than that of CsPbBr_3_ nanocrystals. The co‐atomic two‐phase interface of 113–125 PNs can significantly influence the Coulomb interactions between electron‐hole pairs through interfacial electronic coupling, facilitating the transfer of electrons and holes and reducing the likelihood of non‐radiative recombination. The extended carrier lifetime enhances their potential to participate in photocatalytic reactions before recombination, thereby promoting the photocatalytic process. Photocurrent response measurements (Figure 2i) were conducted to further explore the variations in the photocurrent response of 113–125 PNs. The number of generated charge carriers directly determines the intensity of the photocurrent. Compared to CsPbBr_3_ nanocrystals, 113–125 PNs display a stronger photocurrent signal. This increased photocurrent is attributed to the identical atomic arrangements at the two‐phase interface of 113–125 PNs, which reduces the interfacial potential barrier for charge transfer and promotes the separation of photo‐generated electrons and holes. With the increase in the number of light exposure cycles, both CsPbBr_3_ nanocrystals and 113–125 PNs exhibit a declining trend in photocurrent. The decline in photocurrent density is very common during photocurrent measurements, as the initial electrochemical reaction places the material in a non‐steady state. With continued illumination, the material gradually stabilizes, leading to the initial decline in photocurrent density.^[^ ^7^, ^18^ ^]^ Overall, these microscale electronic characterizations indicate that the strengthened electronic interaction within the lattice‐matched co‐atomic interface of 113–125 PNs notably enhances the interfacial electronic coupling, thus overcoming interfacial charge transfer barriers and promoting charge transfer. These advantages will collectively contribute to ultimately enhancing the photocatalytic performance.

### 2.4 Co‐Atomic Interface Reinforced Electron Transition Process

**Figure 3:**
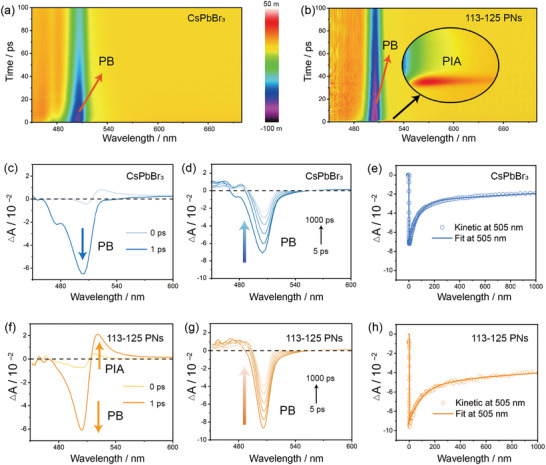
Interface‐enhanced charge recombination in the 113–125 PNs. Pseudocolor TAS of a) CsPbBr_3_ and b) 113–125 PNs. Comparative PIA and PB growth dynamics of c) CsPbBr_3_ and f) 113–125 PNs; PB recovery dynamics of d) CsPbBr_3_ and g) 113–125 PNs. Ultrafast TAS kinetics and the fitting results for e) CsPbBr_3_ and h) 113–125 PNs (at 505 nm).

We employed the TAS technique to investigate the ultrafast carrier relaxation dynamics of CsPbBr_3_ nanocrystals and 113–125 PNs. This study helped to further clarify the electron transfer pathways in 113–125 PNs. TAS measurements were conducted under pulsed excitation at 400 nm, with an excitation intensity of 25 µJ cm^−2^, probing the range between 450 and 700 nm, the vertical axis △A (ΔAbsorbance) in the figure represents the change in absorbance. **Figure** **3**a,b depict the 2D pseudocolor TAS plots for CsPbBr_3_ nanocrystals and 113–125 PNs, respectively. Both samples demonstrate the growth and recovery processes of the photobleach (PB) band ≈505 nm upon optical pumping. The main source of the PB signal is the excited states or generated charge carriers. The more negative the PB signal, the greater the number of generated charge carriers. It can be obviously seen that 113–125 PNs generate significantly more carriers than CsPbBr_3_ nanocrystals. However, as illustrated in the magnified view in Figure 3b, in terms of the 113–125 PNs, there is a significantly stronger photoinduced absorption (PIA) band in the TAS signal. The source of PIA signal is the generation of a new absorption state. This observation indicates the presence of additional carrier relaxation pathways in the 113–125 PNs, which could be originated from the lattice‐matched co‐atomic interface.^[^ ^19^ ^]^ Figure 3c,f illustrate the evolution of transient bands within the delay range of 0 to 1 ps. The PIA band of CsPbBr_3_ decays rapidly within 1 ps, indicating the prompt thermal radiation recombination of rapidly generated charge carriers after excitation. This thermal radiation recombination is achieved through charge recombination in surface traps (fast) and bulk traps (slow) states.^[^ ^20^ ^]^ In contrast to the transients observed in CsPbBr_3_ nanocrystals, the evolution rate of the PIA band in the 113–125 PNs is significantly slower. We attribute the PIA band in 113–125 PNs to the Stark effect induced by excitons, causing an early delay due to the Coulomb interaction between hot excitons and band‐edge excitons.^[^ ^21^ ^]^ In simpler terms, the charge transfer at the co‐atomic interface of 113–125 PNs can efficiently slow down the charge recombination induced by thermal radiation. Additionally, Figure 3d,g illustrate the recovery processes of the PB signals for CsPbBr_3_ and 113–125 PNs, respectively. After the initial intensity of the PB signal increases to its maximum value, the strong PB signal as the hot charge carriers relax to the band‐edge state with the lowest energy and eventually recovers. We observe that within the 505 nm range, the negative amplitude of the PB signal in 113–125 PNs is significantly stronger than that in CsPbBr_3_ nanocrystals, indicating an increase in photoexcited charge carriers in 113–125 PNs. The increased charge carriers further suggest that the co‐atomic structure at the interface of 113–125 PNs can effectively dissociate excitons, resulting in the generation of more electrons and holes.

We further compared the dynamics of CsPbBr_3_ and 113–125 PNs to characterize the electron transfer pathways. Under the excitation wavelength of 505 nm, CsPbBr_3_ exhibits decay characteristics in both time constant and amplitude (Figure 3e). The transient absorption decay can be well‐fitted with two exponential components: τ_1_ = 1.6 ns and τ_2_ = 73.93 ps. The longer carrier lifetime (1.6 ns) corresponds to the radiative recombination of electrons and holes in CsPbBr_3_. The shorter lifetime (73.93 ps) may arise from the radiative process of surface trap states.^[^ ^22^ ^]^ In contrast to CsPbBr_3_, the dynamics curve of 113–125 PNs exhibits a significantly enhanced amplitude (Figure 3h), indicating a more pronounced electron transport process. Simultaneously, due to the presence of multiple phases, a more complex carrier dynamic is observed, with three distinct lifetimes: τ_1_ = 4.712 ns, τ_2_ = 684.8 ps, and τ_3_ = 95.53 ps. The longest lifetime (4.712 ns), compared to CsPbBr_3_ (τ_1_ = 1.6 ns), suggests efficient carrier separation and prolonged recombination time scales. The shortest lifetime (95.53 ps) observed in 113–125 PNs may be attributed to surface defects or trap‐assisted recombination at the interface. The intermediate lifetime (684.8 ps) represents additional energy transfer and carrier relaxation processes in 113–125 PNs, where the coexistence of CsPbBr_3_ and CsPb_2_Br_5_ provides opportunities for efficient carrier transfer. Multiple phases result in a range of carrier lifetimes, signifying varied recombination rates and transfer mechanisms. The heterojunction between CsPbBr_3_ and CsPb_2_Br_5_ introduces additional carrier transfer pathways across the lattice‐matched co‐atomic interface, potentially enhancing charge separation and transfer efficiency. Moreover, the co‐atomic interface in 113–125 PNs may facilitate the creation of charge gradients, favoring charge migration toward specific active sites. Finally, the broader range of carrier lifetimes observed suggests a more intricate network of energy and charge transfer pathways, which can contribute to enhanced photocatalytic CO_2_ reduction efficiency.

### 2.5 Photocatalytic CO_2_ Reduction Performance

**Figure 4:**
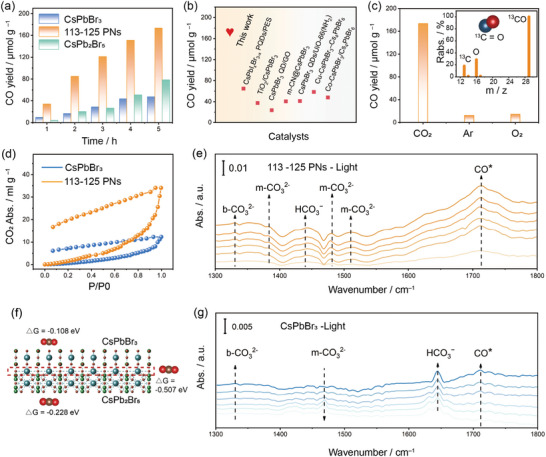
Photocatalytic CO_2_ reduction performance evaluation and mechanism. a) Time‐yield plots of CO on CsPbBr_3_, 113–125 PNs, and CsPb_2_Br_5_. b) Comparison of the photocatalytic activity over various CsPbBr_3_‐based heterojunction photocatalysts. c) Photocatalytic performance under different reaction conditions and inset is the mass spectra of CO generated under ^13^CO_2_ atmosphere of 113–125 PNs. d) CO_2_ adsorption evaluation on CsPbBr_3_ and 113–125 PNs at 298 K. f) The calculated free energy of CO_2_ adsorption process for 113–125 PNs. In situ DRIFTS test of CO_2_ interaction with e) 113–125 PNs and g) CsPbBr_3_ under constant simulated solar illumination.

We evaluated the photocatalytic performance of CO_2_ reduction in a gas‐solid reaction system under AM 1.5G simulated solar illumination without employing co‐catalysts or sacrificial reagents. Prior to the activity test, the residual solvent molecules on the sample surface are purified by vacuum drying for 24 h to minimize the pollution. **Figure** **4**a presents the activity data for each hour of the photoreduction process of 113–125 PNs, CsPbBr_3_, and CsPb_2_Br_5_. CO is the main gaseous product. The rest of the products, such as H_2_ and CH_4_, are negligible. It can be observed that the CO yield rate of 113–125 PNs remained stable in each test time, indicating that 113–125 PNs have excellent photocatalytic stability. After 5 h irradiation, 113–125 PNs exhibited the highest CO production rate of 173.3 µmol g^−1^, nearly 3.6 times higher than that of pure CsPbBr_3_ nanocrystals. Besides, this rate surpassed that of CsPb_2_Br_5_ and post‐assembled CsPbBr_3_+CsPb_2_Br_5_ by 2.2 and 2.8 times, respectively (Figures S8 and S9, Supporting Information). The difference in activity between 113–125 PNs and the post‐assembled CsPbBr_3_+CsPb_2_Br_5_ further validates the role of co‐atomic interfaces in lowering charge transfer barriers and facilitating efficient charge transfer processes. More importantly, we conducted a 20 h stability test on 113–125 PNs to ensure that it is a reliable photocatalyst. As shown in Figure S10 (Supporting Information), compared to CsPbBr_3_ nanocrystals, 113–125 PNs maintained stable activity growth with almost no activity decay. The 20 h cumulative CO yield of 113–125 PNs reached 607 µmol g^−1^, which is 7.45 times that of CsPbBr_3_ nanocrystals. The stable activity validates that 113–125 PNs is a qualified photocatalyst. To evaluate the stability of 113–125 PNs under experimental conditions, we performed XRD and UV–vis DRS tests before and after the photocatalytic reaction. The results, shown in Figures S11 and S12 (Supporting Information), indicate that there were no changes in the phase of 113–125 PNs after the reaction, and the optical absorption edge and intensity remained stable. This demonstrates that both the structural and optical absorption properties of the material remained unchanged after the reaction, ensuring the stability of 113–125 PNs under experimental conditions. To verify the recyclability of 113–125 PNs, we redissolved the catalyst loaded on glass fiber in toluene. The initial loading was 9.8 mg, but after reloading, there was some loss, resulting in a catalyst content of 6.8 mg, while maintaining activity similar to that of the original 113–125 PNs. (Figure S13, Supporting Information). Additionally, compared to other CsPbBr_3_‐based heterojunction photocatalysts, the 113–125 PNs also exhibit exceptionally high CO yield, as summarized in Table S1 (Supporting Information) and Figure 4b. Therefore, it can be inferred that 113–125 PNs have significant advantages for CO_2_ photoreduction. This excellent performance is attributed to the co‐atomic interface in 113–125 PNs, which, in contrast to common heterojunction structures, greatly strengthens the interfacial electronic coupling and reduces the interfacial charge transfer barriers, thereby facilitating efficient charge separation and transfer. We conducted a series of control experiments to mitigate the influence of impurities on photocatalytic activity. As displayed in Figure 4c, only a small amount of CO product was produced under a pure Ar and O_2_ atmosphere, likely originating from trace solvent residues. This result underscores the effectiveness of the gas‐solid phase photocatalytic reaction in mitigating pollution from residual organic impurities. Besides, isotopically labeled tests (^13^CO_2_) were also performed to verify the production of CO derived from CO_2_ reduction. As shown in the inset of Figure 4c, the fragment (^13^C at *m/z* = 13 and ^13^CO at *m/z* = 29) produced in the mass spectrum demonstrates that the produced ^13^CO is directly derived from ^13^CO_2_.^[^ ^23^ ^]^ Collectively, all evidence indicates that 113–125 PNs can efficiently catalyze the photocatalytic reduction of CO_2_ to CO in gas‐solid phase reactions.

### 2.6 Mechanistic Investigation of CO_2_ Photoreduction

To elucidate the reasons for the enhanced photocatalytic CO_2_ reduction activity of polymorphic nanocrystals and to investigate the CO_2_ photoreduction mechanism of 113–125 PNs, along with the contribution of co‐atomic interfaces to the catalytic system, we conducted CO_2_ adsorption experiments on CsPbBr_3_ and 113–125 PNs, as shown in Figure 4d. The results indicate that the CO_2_ adsorption capacity of 113–125 PNs is more vital than that of CsPbBr_3_. This result suggests that CO_2_ can be more efficiently adsorbed on the catalyst surface equipped with the lattice‐matched co‐atomic interface, thereby creating favorable conditions for subsequent CO_2_ photocatalytic reduction reactions. Additionally, we calculated the adsorption energies of various CO_2_ adsorption sites, as illustrated in Figure 4f. The adsorption energies on the surfaces of CsPbBr_3_ and CsPb_2_Br_5_ are −0.108 eV and −0.228 eV, respectively, indicating that these individual surfaces are not favorable for CO_2_ adsorption. In contrast, at the common interface of 113–125 PNs, the CO_2_ adsorption energy is −0.507 eV, suggesting significantly stronger CO_2_ adsorption at this interface. This increased adsorption is attributed to the lattice‐matched co‐atomic interface, which reduces the electronic transfer barrier. Consequently, electrons within the 113–125 PNs possess sufficient kinetic energy to overcome the binding of surface‐adsorbed molecules, leading to the formation of intermediates with higher adsorption energies. These intermediates enhance the efficiency of subsequent electron transfer, thereby significantly increasing the photocatalytic CO_2_ reduction activity.

For a more precise tracking of the activation process of H_2_O and CO_2_ further precisely on the surface of 113–125 PNs, we employed the in situ diffuse reflectance infrared Fourier transform spectroscopy (DRIFTS) to investigate the photocatalytic mechanism of CO_2_ reduction on both the CsPbBr_3_ phase and the 113–125 PNs. The evolution of surface‐bound species on the sorption‐equilibrated CsPbBr_3_ phase and 113–125 PNs catalysts under constant irradiation was observed. As shown in Figure 4e, the in situ DRIFTS of 113–125 PNs under simulated sunlight irradiation reveal multiple reaction intermediate products. The detected surface species are summarized in Table S2 (Supporting Information). The peak at 1331 cm^−1^ corresponds to bidentate carbonate (b‐CO_3_^2−^),^[^ ^24^ ^]^ while the peaks at 1385, 1481, and 1510 cm^−1^ correspond to monodentate carbonate (m‐CO_3_^2−^),^[^ ^25^ ^]^ representing two forms of CO_2_ adsorption. Additionally, the peaks at ≈1430–1440 cm^−1^ are attributed to bicarbonate (HCO_3_^−^), suggesting that during the photocatalytic reduction process, CO_2_ is activated to generate intermediate species such as carbonate. Worth noting is a peak at 1711 cm^−1^, possibly associated with the bending vibration of C═O in produced CO.^[^ ^26^ ^]^ The appearance of characteristic CO* peaks indicates the successful photocatalytic reduction of CO_2_ to CO by 113–125 PNs. Similarly, the intermediate species produced from CO_2_ activation under light irradiation for CsPbBr_3_ nanocrystals (Figure 4g) is identical to that of 113–125 PNs, suggesting a potentially similar reaction pathway for these two samples (CO_2_→CO_2_^−^→HCO_3_^−^→CO). However, the CsPbBr_3_ exhibits weaker intensity and less significant variations in all spectral bands, and the surface adsorbs m‐CO_3_^2−^ on the CsPbBr_3_ do not accumulate but gradually deplete under light. The disappearance of intermediate products demonstrates that there is not enough electron transfer from the surface of CsPbBr_3_ to CO_2_, further hindering subsequent catalytic conversion. The in situ DRIFTS results indicate that 113–125 PNs maintain the same CO_2_ reduction pathway as CsPbBr_3_. However, the presence of lattice‐matched co‐atomic interfaces reduces the charge transfer barrier. This modulated barrier allows sufficient electron accumulation at the interface of 113–125 PNs to adsorb CO_2_ and transfer electrons to CO_2_, catalyzing its conversion. Therefore, the reduced charge transfer barrier at the co‐atomic interface is a key factor contributing to the excellent photocatalytic CO_2_ reduction efficiency of 113–125 PNs.

## 3 Discussion

In this study, we created a lattice‐matched co‐atomic interface within 113–125 PNs using an in situ method to overcome the high interfacial barrier between the two phases of traditional heterojunction structures caused by lattice mismatch. The results indicate that the photocatalytic CO_2_ reduction efficiency of 113–125 PNs with the co‐atomic interface is 3.63 times higher than that of pure phase CsPbBr_3_. The consistent arrangement of co‐atoms at the 113–125 PNs interface significantly promotes the interfacial electronic coupling and reduces the interfacial charge transfer barrier. Furthermore, the co‐atomic interface offers supplementary locations for carrier generation and separation, enhancing the inherent electric field and establishing a distinct pathway for charge transfer. This charge transfer channel, in turn, significantly boosts the efficiency of charge transfer and utilization. This study provides new insights into optimizing the interfacial structures, exploring the co‐catalyst integration, and advancing the understanding of interface charge transfer processes.

## 4 Experimental Section

### Materials

Cesium carbonate (Cs_2_CO_3_, 99%), lead bromide (PbBr_2_, 99%), Oleylamine (OAm, 90%), Oleic Acid (OA, 90%) were acquired from Aladdin Chemical Reagent Co., Ltd (Shanghai, China). 1‐Octadecene (ODE, 90%) was purchased from Adamas Reagents. Toluene and acetone were all obtained from Sinopharm Chemical Reagent Co., Ltd (Shanghai, China). All the chemicals were of analytical purity and used without further purification.

### Preparation of CsPbBr_3_ Nanocrystals and 113–125 PNs

All reaction processes were carried out under Ar protection. CsPbBr_3_ nanocrystals were synthesized by hot‐injection method following the previous publications, and the sample named CsPbBr_3_ nanocrystals. 113–125 PNs synthesized on the basis of successfully synthesized CsPbBr_3_ nanocrystals. The cooled solution of CsPbBr_3_ Nanocrystals was heated again to 210 °C and held for 5 min, and the solution was put into an ice‐water mixture to make the solution temperature drop rapidly to 40 °C to end the whole reaction. The washing and centrifugation process was the same as that of CsPbBr_3_ Nanocrystals, and the final obtained 113–125 PNs.

### Characterization

A series of characterizations were effectuated to explore the relevant characteristics of 113–125 PNs. XRD patterns with CuKα radiation (Model D/max RA, Rigaku Co.) were carried out to obtain crystal phases. XPS (Thermo ESCALAB 250) with an Al Kα (1486.6 eV) radiation source was performed to analyze the surface chemical state. The size and morphology of the photocatalysts were observed by TEM (Tecnai G2 F20 S‐TWIN, FEI). UV–vis DRS were measured upon a UV‐visible near infrared spectrophotometer (UV–2450, Shimadzu) with a diffuse reflectance accessory to detect optical absorption properties. Steady and time‐resolved fluorescence emission spectra were recorded at room temperature with a fluorescence spectrophotometer (Edinburgh Instruments, FLS1000). KPFM was obtained to analyze the surface potential of materials by atomic force microscopy (AFM, Bruker Icon). The TAS data were recorded on a femtosecond pump‐probe spectrometer (Ultrafast Systems, Helios Fire) coupled to an ultrafast amplified laser system (Spectra–Physics, Solstice ACE). The in situ DRIFTS equipment with a Nicolet iS50FT‐IR spectrometer (Thermo Fisher, USA) instrument was equipped with a tailor‐made reactor and liquid nitrogen‐cooled HgCdTe (MCT) detector.

### Photocatalytic Activity Testing

The performance of the photocatalytic CO_2_ reduction was evaluated on the MC‐SC0211‐AG system (Beijing MerryChange Technology Co., Ltd.), which assisted with a gas chromatography (GC) instrument for product analysis, and a 300 W Xe lamp equipped with AM 1.5G filter was used to simulate full‐spectrum sunlight. The average intensity was determined by an optical power meter (CEL‐NP2000, Au‐light) to be 88.3 mW cm^−2^. Typically, 10 mg of photocatalyst was ultrasonically dispersed in a small amount of toluene, and the solution was evenly distributed on a glass fiber membrane with a pore size of 0.25 µm. Then the sample membrane was placed in a vacuum oven that was continuously evacuated for a whole day and night to remove excess toluene solution thoroughly. Afterward, the glass fiber membrane was transferred into a fully enclosed quartz reactor, and the reactor temperature was maintained at 20 °C by circulating water through the reactor to eliminate the thermal effect of radiation. Pure CO_2_ with a trace of water vapor was introduced into the reactor to thoroughly sweep the residual O_2_ and other foreign gases. The gaseous products were analyzed online with a GC instrument equipped with a flame ionization detector (FID) and a thermal conductivity detector (TCD) every hour.

### Electrochemical Measurements

The electrochemical test was carried out on the electrochemical workstation (CHI660E, Shanghai) in the standard three‐electrode system with Pt net as the counter electrode and calomel electrode as the reference electrode. Prior to the test, the working electrode was prepared by grinding 10 mg of the photocatalyst powder with 2 mL of toluene in an agate mortar to create a plating solution. The resulting solution was then uniformly deposited onto the FTO substrate and dried in a vacuum oven at 393 K for 2 h. The coating area of all samples was carefully controlled at 1 cm^2^ during electrochemical measurement. The electrolyte was 0.1 M tetrabutylammonium hexafluorophosphate (TBAPF6) ethyl acetate solution.

### Isotope Labeling Experiment

The isotope labeling experiment was conducted under the same reaction conditions used for photocatalytic CRR evaluation, where the pure ^12^CO_2_ gas was substituted with ^13^CO_2_ gas. The gaseous of ^13^CO products were monitored using gas chromatography–mass spectrometry (GC‐MS), where a Shimadzu GC‐2010 GC instrument equipped with a chromatographic column (30 m × 0.32 mm × 25 µm; HP‐MOLESIEVE, Agilent Technologies, USA) was coupled to a Shimadzu QP2010 mass spectrometer.

### DFT Calculation Details

The spin‐polarized DFT calculation was carried out by Vienna ab initio simulation package (VASP5.4.1). The generalized gradient approximation with the Perdew−Burke−Ernzerhof (PBE) exchange and correlation functional was utilized. A 3 × 3 × 1 K‐point was sampled in the Brillouin zone. A plane wave energy cutoff of 420 eV was used to optimize the geometric structures, and the energy and electronic forces were converged to 1 × 10^−4^ eV and 0.03 eV Å^−1^, respectively. The Gibbs free energies in CO_2_ adsorption were calculated at 298.15 K, and the calculation formula is defined as △G = E_DFT_ − TS + E_ZPE_, where E_DFT_, TS, and E_ZPE_ stand for the DFT energy, entropy contribution, and zero‐point energy, respectively.

## Conflict of Interest

The authors declare no conflict of interest.

## Supporting information



Supporting Information

## Data Availability

The data that support the findings of this study are available from the corresponding author upon reasonable request.
