# Supplementary material for: Co‐Atomic Interface Minimizing Charge Transfer Barrier in Polytypic Perovskites for CO2 Photoreduction
Source: Adv Sci (Weinh). 2025 Jan 10;12(9):2410437. doi: 10.1002/advs.202410437 (PMC11884529; doi:10.1002/advs.202410437)
Supplement: Supplementary file 1 — Supporting Information [file ADVS-12-2410437-s001.docx]

**Co-Atomic Interface Minimizing Charge Transfer Barrier in Polytypic Perovskites for CO_2_ Photoreduction**

*Fengyi Zhong, Jianping Sheng,* Chenyu Du, Ye He, Fengying Zhang, Yanjuan Sun, Ying Zhou, and Fan Dong**

F. Zhong, J. Sheng, C. Du, Y. He, Y. Sun, F. Dong

School of Resources and Environment, University of Electronic Science and Technology of China, Chengdu 611731, China
E-mail: jpshengchn@163.com; dfctbu@126.com; [dongfan@uestc.edu.cn](mailto:dongfan@uestc.edu.cn)

J. Sheng, Y. Sun, F. Dong
Institute of Fundamental and Frontier Sciences, University of Electronic Science and Technology of China, Chengdu 611731, China

F. Zhang, Y. Zhou
School of New Energy and Materials, Southwest Petroleum University, Chengdu 610500, China




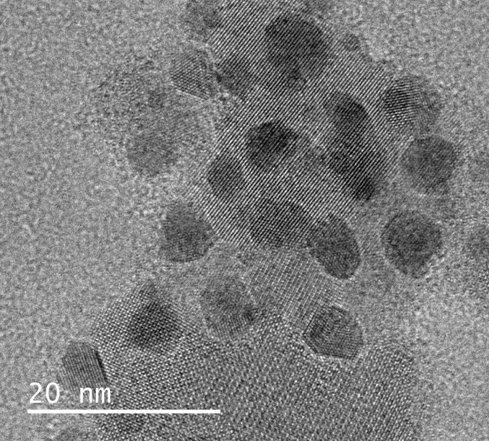


**Figure S1** The TEM of progressive growth of the CsPb_2_Br_5_ phase over time.


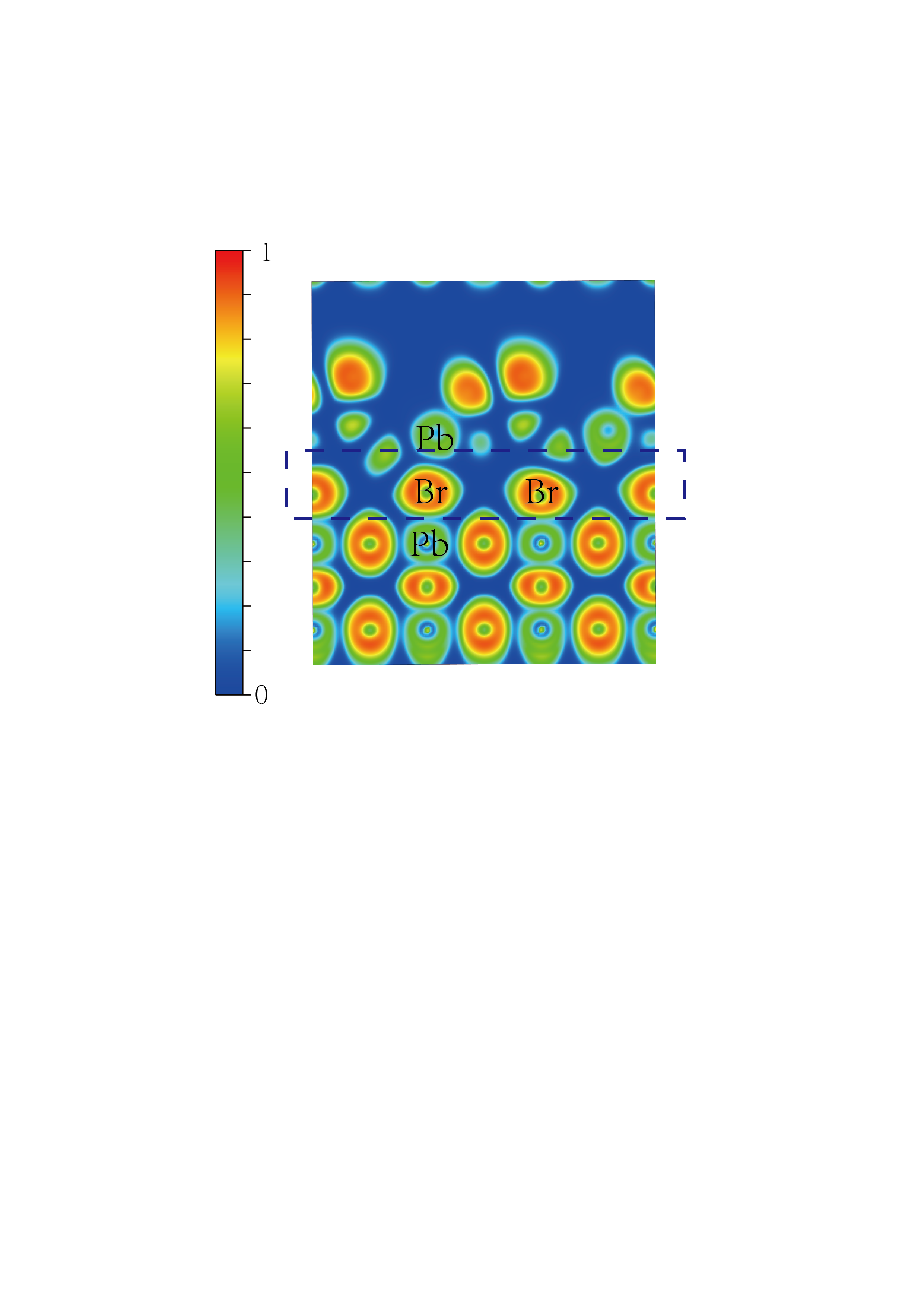


**Figure S2** The electronic location function results of 113-125 PNs.


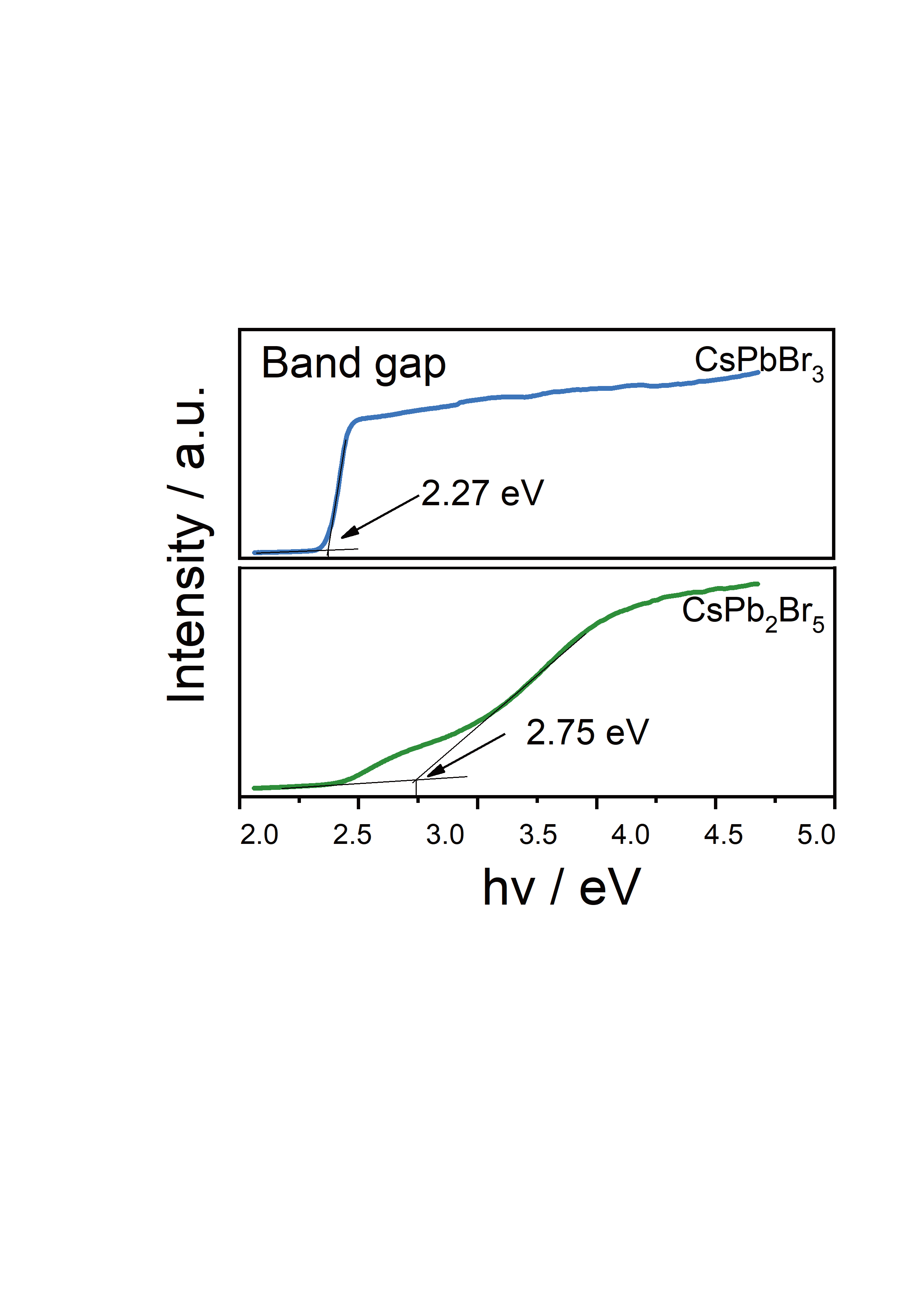


**Figure S3** The bandgap values according to the Tauc plot of CsPbBr_3_ and CsPb_2_Br_5_.


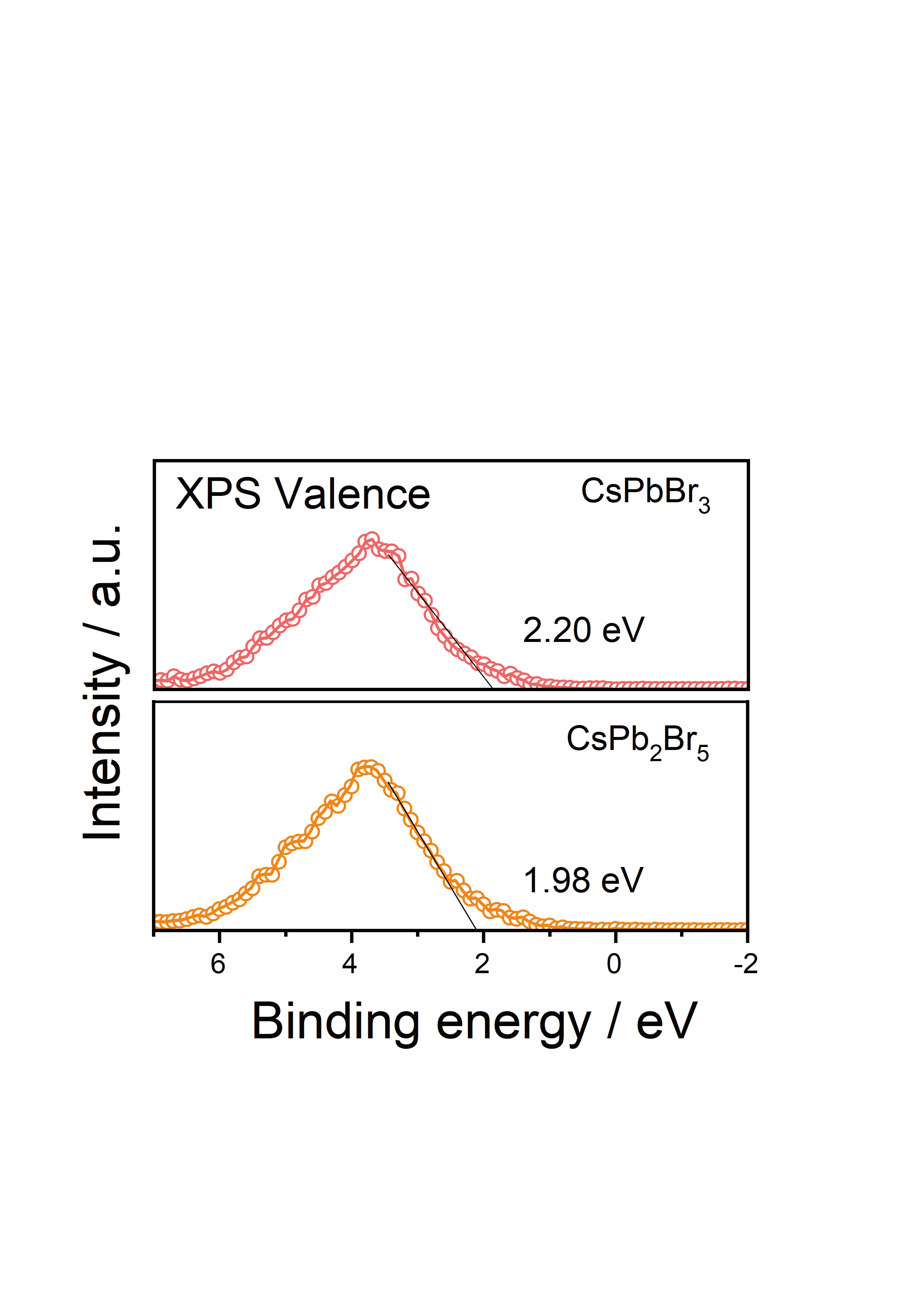


**Figure S4** The valence band of CsPbBr_3_ and CsPb_2_Br_5_.


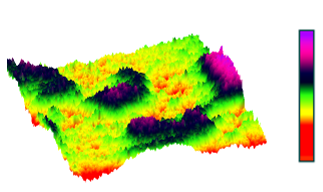


**Figure S5** 3D surface potential distribution of CsPbBr_3_. The scale bar is -50 to 50 mV.


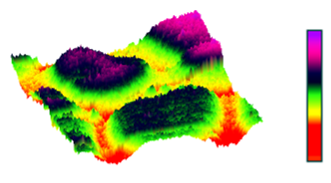


**Figure S6** 3D surface potential distribution of 113-125 PNs. The scale bar is -50 to 50 mV.


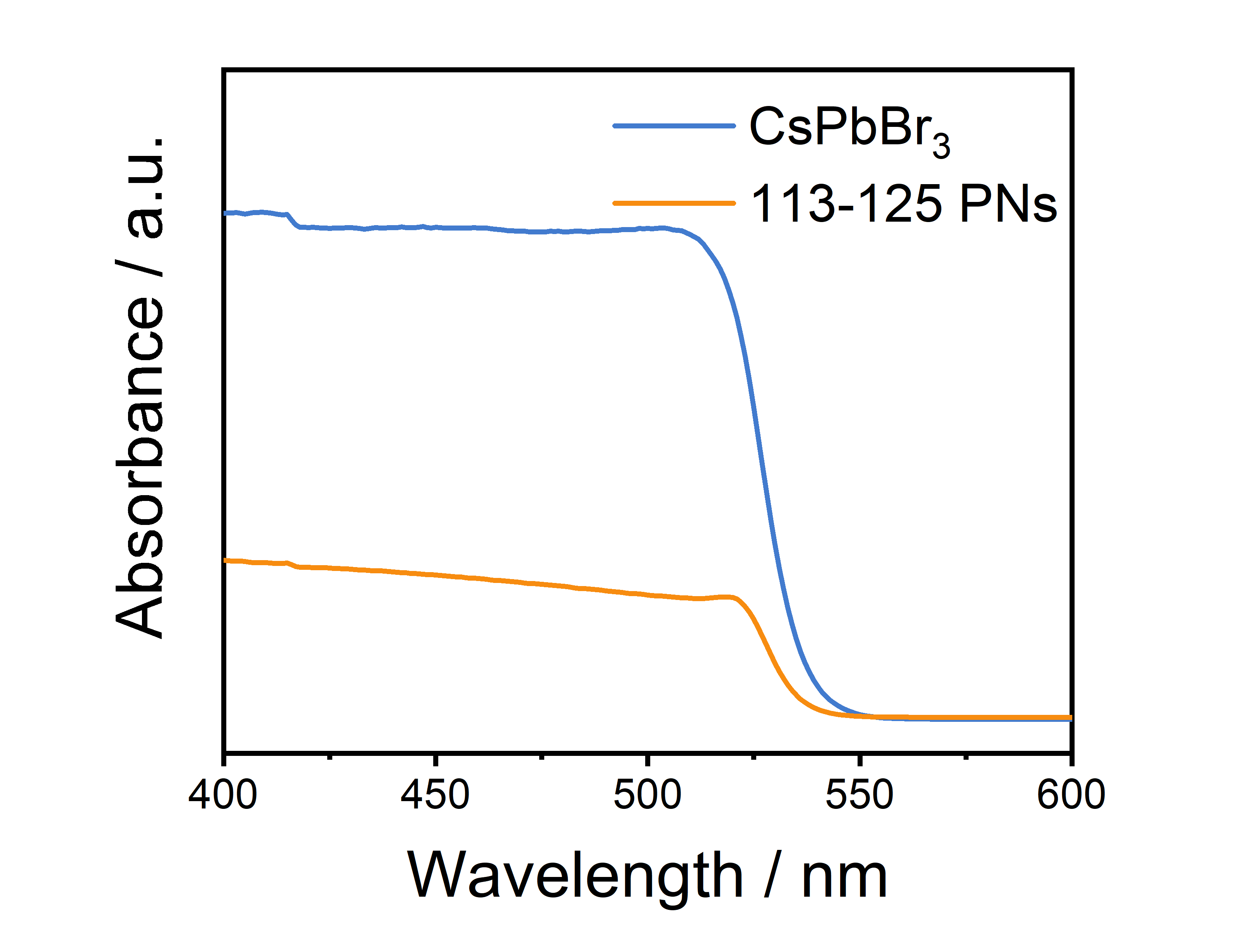


**Figure S7** the UV-vis DRS spectra of CsPbBr_3_ and 113-125 PNs**.**


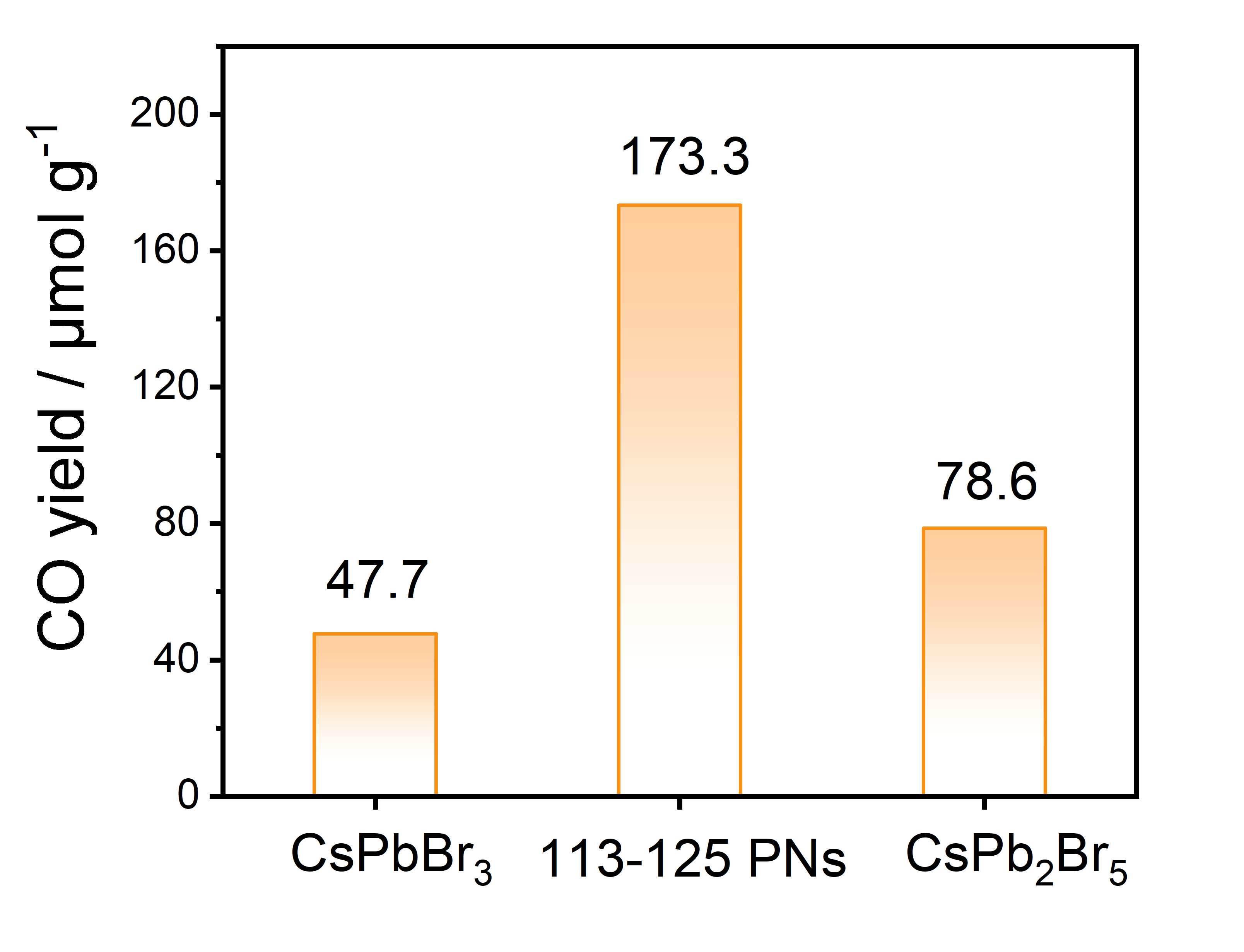


**Figure S8** CO yield of CsPbBr_3_, 113-125 PNs and CsPb_2_Br_5_ after 5 h irradiation.


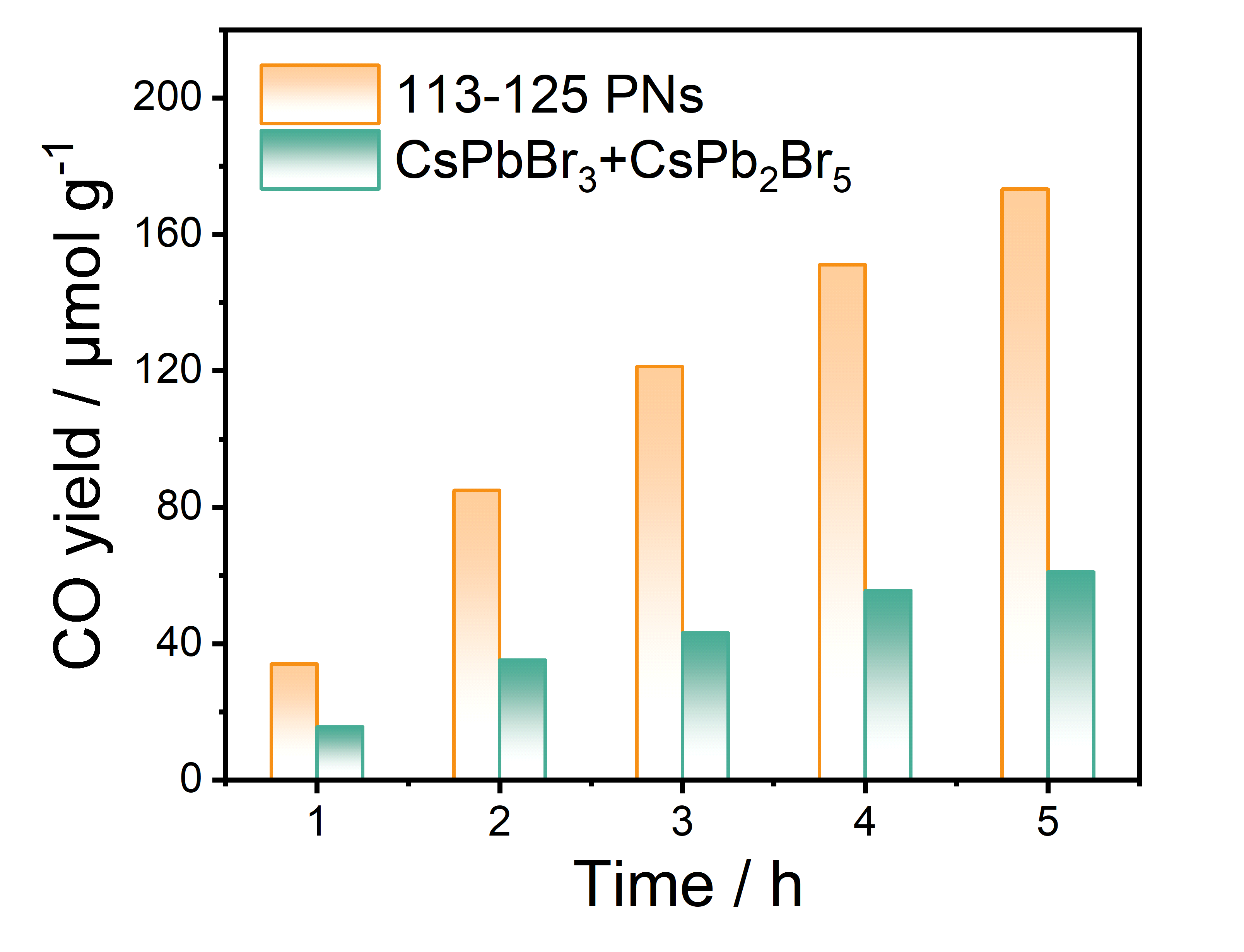


**Figure S9** Time-yield plots of CO of 113-125 PNs and CsPbBr_3_+CsPb_2_Br_5_.


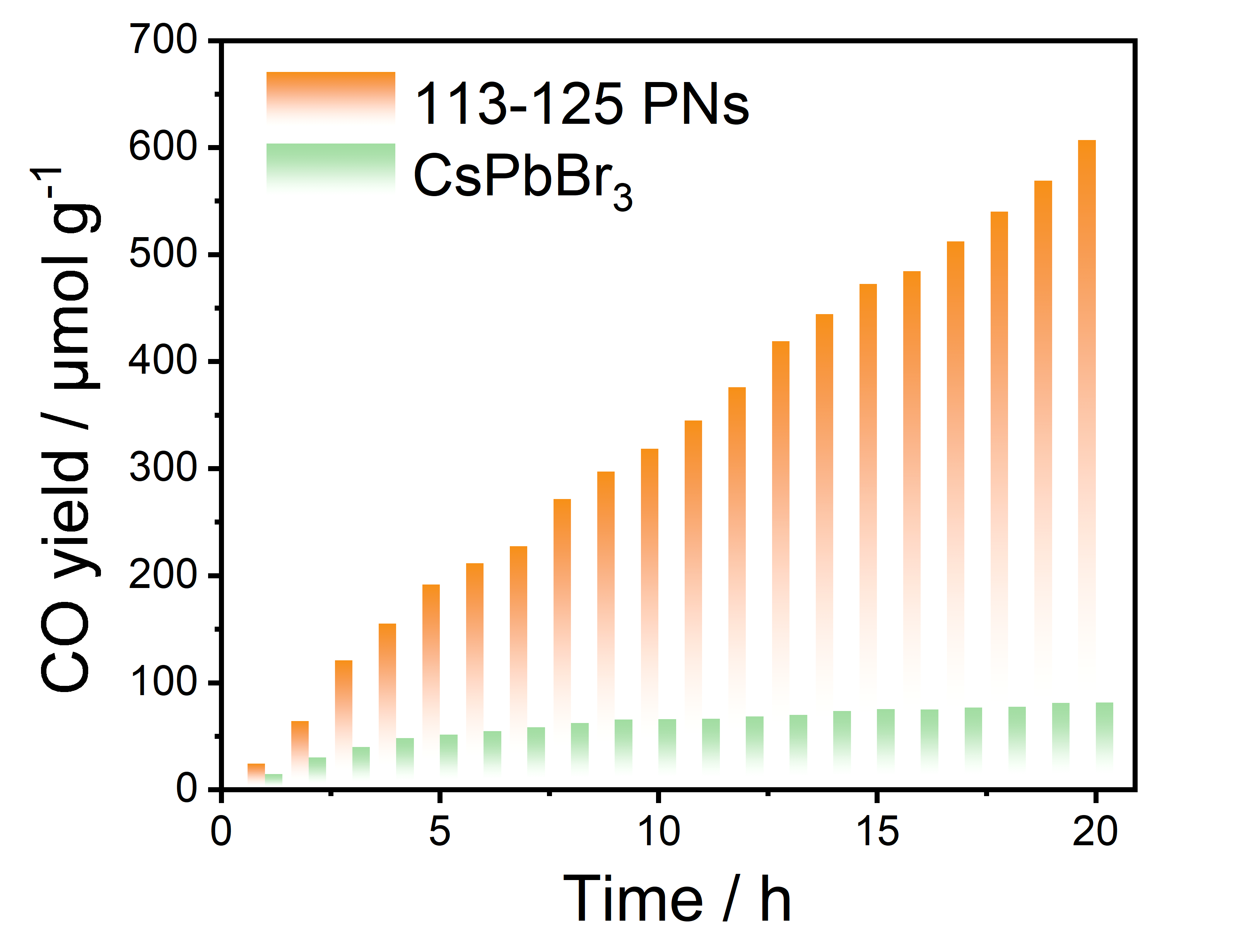


**Figure S10** CO plots over 113-125 PNs and *CsPbBr_3_* for the 20 h stability test.


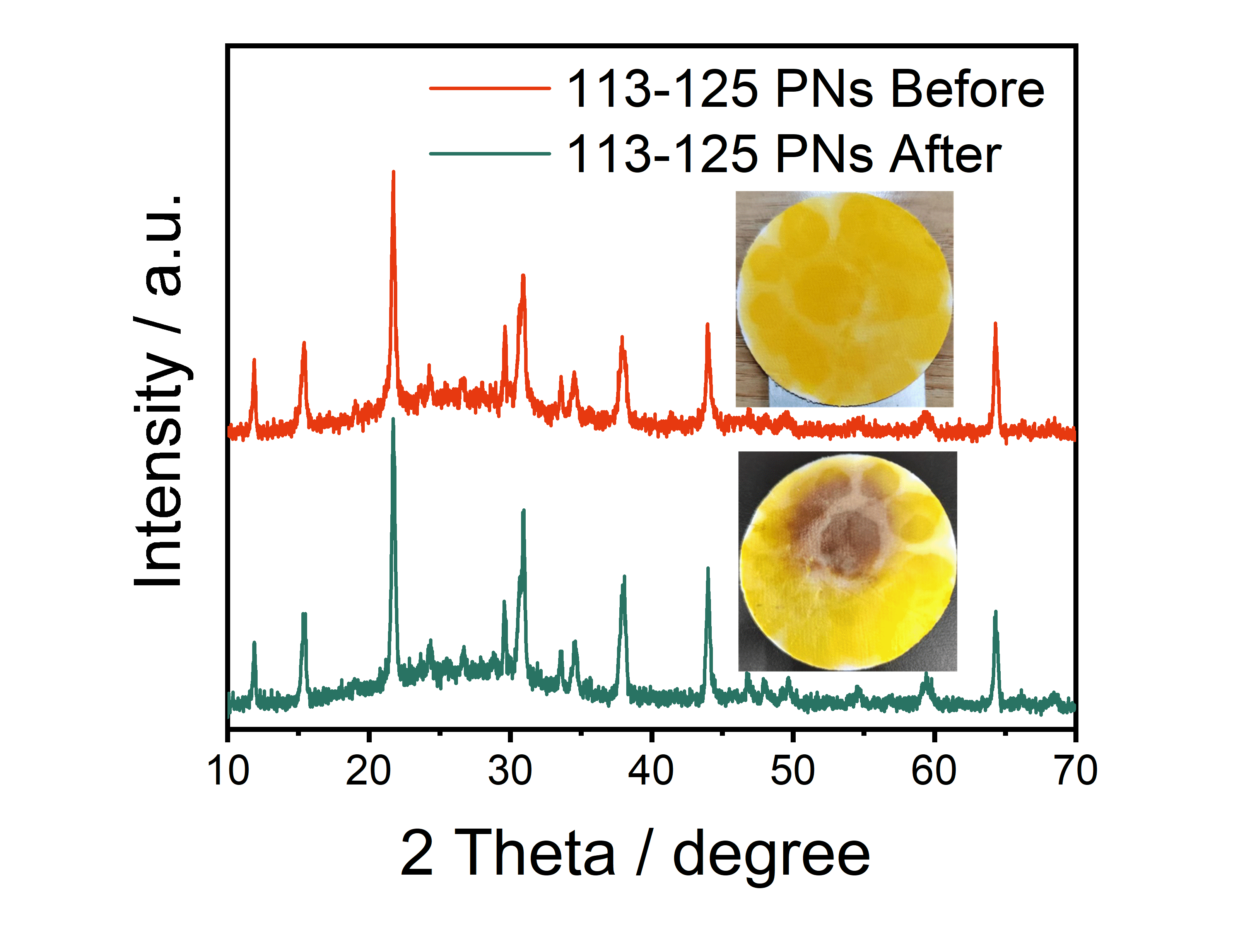


**Figure S11** The XRD spectra of 113-125 PNs before and after the reaction.


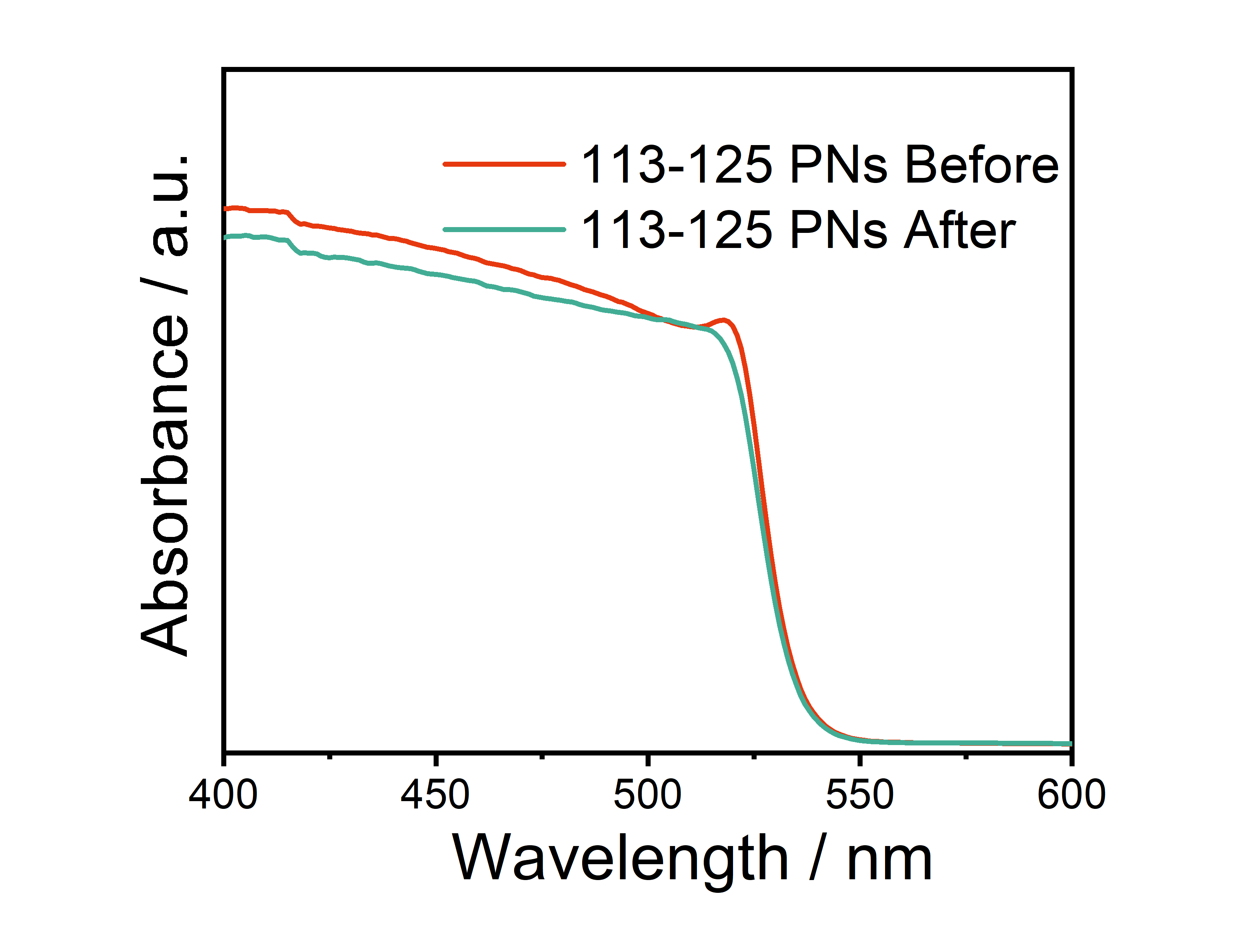


**Figure S12** The UV–vis DRS of 113-125 PNs before and after the reaction.


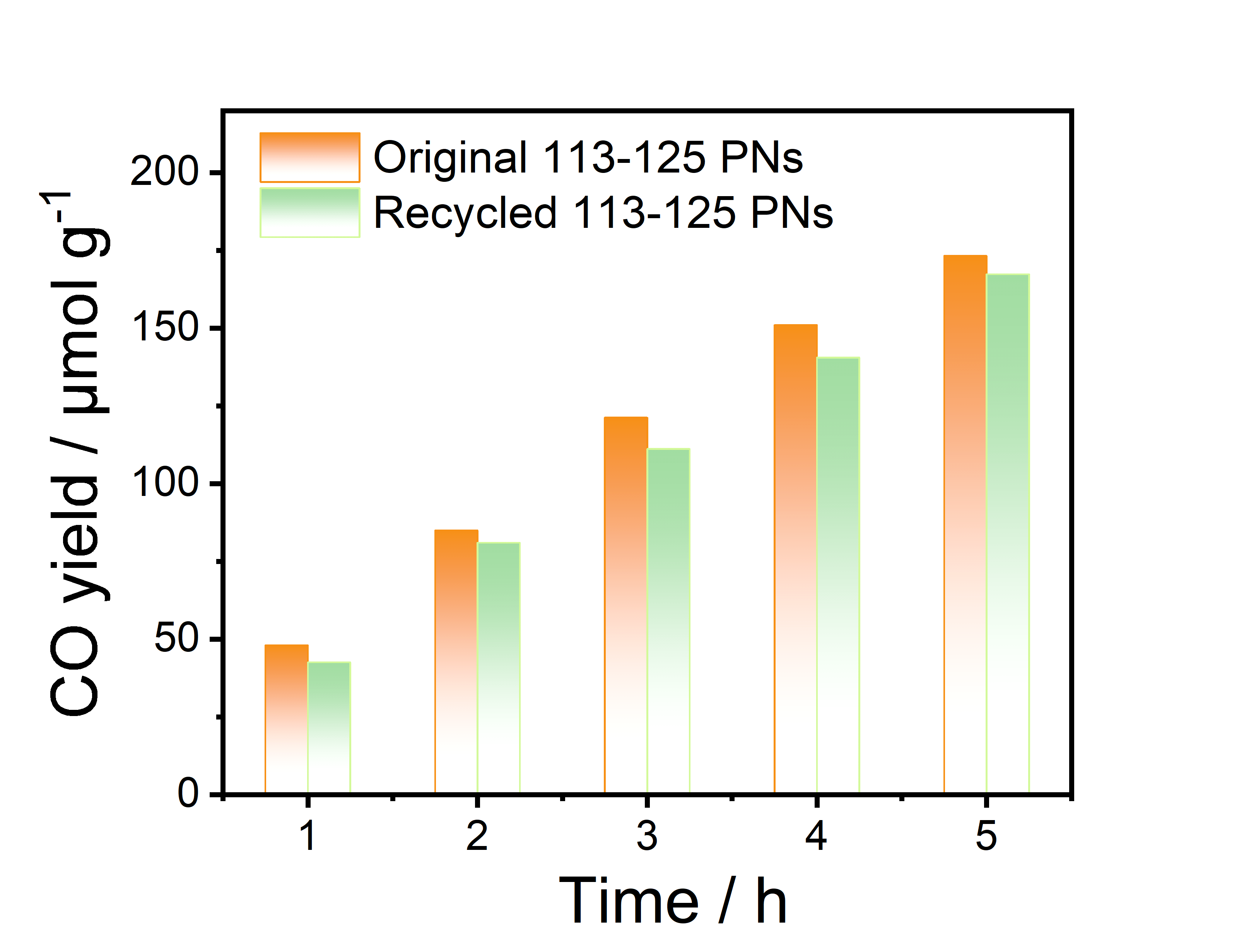


**Figure S13** Time-yield plots of CO on original 113-125 PNs and recycled 113-125 PNs.

**Table S1** Summary of the photocatalytic CO_2_ reducing activity over various CsPbBr_3_-based heterojunction photocatalysts.

| **Catalysts** | **CO μmol g^-1^** | **Ref.** |
| --- | --- | --- |
| 113-125 PNs | 173.3 | This work |
| CsPbI_x_Br_3-x_ PQDs/PES | 64.9 | [1] |
| TiO_2_/CsPbBr_3_ | 37.4 | [2] |
| CsPbBr_3_ QD/GO | 23.7 | [3] |
| m-CN@CsPbBr_3_ | 40.6 | [4] |
| CsPbBr_3_ QDs/UiO-66(NH_2_) | 41.1 | [5] |
| Cu-CsPbBr_3_–Cs_4_PbBr_6_ | 58.5 | [6] |
| Co-CsPbBr_3_/Cs_4_PbBr_6_ | 48.0 | [7] |

# References

[1] R. Cheng, C. C. Chung, S. Wang, B. Cao, M. Zhang, C. Chen, Z. Wang, M. Chen, S. Shen, S. P. Feng, Mater. Today Phys. 2021, 17, 100358.

[2] F. Xu, K. Meng, B. Cheng, S. Wang, J. Xu, J. Yu, Nat. Commun. 2020, 11, 4613.

[3] Y. F. Xu, M. Z. Yang, B. X. Chen, X. D. Wang, H. Y. Chen, D. B. Kuang, C. Y. Su, J. Am. Chem. Soc. 2017, 139, 5660-5663.

[4] H. Bian, D. Li, S. Wang, J. Yan, S. Liu, Chem. Sci. 2022, 13, 1335-1341.

[5] S. Wan, M. Ou, Q. Zhong, X. Wang, Chem. Eng. J. 2019, 358, 1287-1295.

[6] L. Li, Z. Zhang, Chem. Eng. J. 2022, 434, 134811.

[7] Y. F. Mu, W. Zhang, X. X. Guo, G. X. Dong, M. Zhang, T. B. Lu, ChemSusChem 2019, 12, 4769-4774.

Table S2. Assigned surface species of the characteristic wavenumbers on113-125 PNs.

| **Wavenumber / cm^-1^** | **Species** | **Ref.** |
| --- | --- | --- |
| 1331 | b-CO_3_^2−^ | [1] |
| 1385, 1481, and 1510 | c-CO_3_^2−^ | [2] |
| 1430-1440 | HCO_3_^−^ | [3] |
| 1716 | CO* | [4,5] |

Ref.

[1] J. Wu, X. Li, W. Shi, P. Ling, Y. Sun, X. Jiao, S. Gao, L. Liang, J. Xu, W. Yan, C. Wang, Y. Xie, Angew. Chem. Int. Ed. Engl. 2018, 57, 8719-8723.

[2] M. Wang, M. Shen, X. Jin, J. Tian, M. Li, Y. Zhou, L. Zhang, Y. Li, J. Shi, ACS Catal. 2019, 9, 4573-4581.

[3] L. Liu, Y. Jiang, H. Zhao, J. Chen, J. Cheng, K. Yang, Y. Li, ACS Catal 2016;6:1097-1108.

[4] X. Yu, Z. Yang, B. Qiu, S. Guo, P. Yang, B. Yu, H. Zhang, Y. Zhao, X. Yang, B. Han, Z. Liu, Angew. Chem. Int. Ed. Engl. 2019, 58, 632-636.

[5] J. Sheng, Y. He, M. Huang, C. Yuan, S. Wang, F. Dong, ACS Catal. 2022;12:2915-2926.

Author Contributions

J. Sheng and F. Dong conceived the research.

J. Sheng, Y. Sun, Y. Zhou, and F. Dong supervised the project.

F. Zhong, C. Du, Y. He, F. Zhang, and Y. Zhou prepared photocatalysts and conducted all the experiments.

F. Zhong performed the photocatalytic activity measurement.

F. Zhong, J. Sheng, and F. Dong wrote and revised the manuscript.

All authors provided critical feedback and assisted during manuscript preparation.
